# Short and Long Term Repeatability of Saccharin Transit Time in Current, Former, and Never Smokers

**DOI:** 10.3389/fphys.2020.01109

**Published:** 2020-09-18

**Authors:** Rosalia Emma, Pasquale Caponnetto, Fabio Cibella, Massimo Caruso, Gianluca Conte, Francesca Benfatto, Salvatore Ferlito, Alessandro Gulino, Riccardo Polosa

**Affiliations:** ^1^Centre for the Prevention and Treatment of Tobacco Addiction (CPCT), Teaching Hospital “Policlinico–V. Emanuele”, University of Catania, Catania, Italy; ^2^Center of Excellence for the Acceleration of Harm Reduction (CoEHAR), Department of Clinical and Experimental Medicine, University of Catania, Catania, Italy; ^3^National Research Council, Institute of Biomedicine and Molecular Immunology, Palermo, Italy; ^4^Department of Biomedical and Biotechnological Sciences, University of Catania, Catania, Italy; ^5^Department of Medical, Surgical Sciences and Advanced Technologies “G.F., Ingrassia”, University of Catania, Catania, Italy; ^6^Department of Clinical and Experimental Medicine, University of Catania, Catania, Italy

**Keywords:** smoking, mucociliary clearance transit time, saccharin test, reproducibility, MCC

## Abstract

Smoking progressively damages the efficiency of mucociliary clearance (MCC) defense mechanisms, thus contributing to increased susceptibility to respiratory infections. Prolonged mucociliary clearance transit time (MCCTT) caused by chronic smoking has been investigated by saccharin test, but little data is available about its short- and long-term reproducibility. Moreover, it is not known if MCC impairment can be reversed when stopping smoking. Objective of the study is to investigate and compare short (3 days) and long term (30 days) repeatability of baseline saccharin transit time (STT) among current, former, and never smokers. STT results were analyzed in 39 current, 40 former, and 40 never smokers. Significant (*p* < 0.0001) short-term and long-term repeatability of STT were observed in current (R squared = 0.398 and 0.672, for short- and long-term, respectively) and former smokers (R squared = 0.714 and 0.595, for short- and long-term, respectively). Significant differences in MCCTT were observed among the three study groups (*p* < 0.0001); the median (IQR) MCCTT being 13.15 (10.24–17.25), 7.26 (6.18–9.17), and 7.24 (5.73–8.73) minutes for current, former and never smokers, respectively. Comparison between current smokers and former smokers was significantly different (*p* < 0.0001). There was no significant difference between former and never smokers. The Saccharin test was well tolerated by all participants. We have shown for the first time high level repeatability in both current and former smokers. Moreover, MCC impairment can be completely reversed, former smokers exhibiting similar STT as never smokers. Measurement of STT is a sensitive biomarker of physiological effect for the detection of early respiratory health changes and may be useful for clinical research.

## Introduction

Smoking is an important cause of preventable morbidity and premature mortality globally, mainly due to lung cancer, acute fatal complications of atherosclerotic cardiovascular disease, and chronic obstructive pulmonary disease (COPD) (Centers for Disease Control and Prevention [CDCP], 2004; World health Organization [WHO], 2015).

Chronic exposure to cigarette smoke causes progressive structural damage and functional alterations in the airways, with development of airway epithelial mucus cell hyperplasia with mucus hypersecretion (Cohen et al., 1979; Domagala-Kulawik, 2008), loss of cilia (Sisson et al., 1994; Jacob et al., 2010), and reduced ciliary beating (Simet et al., 2010; Kuehn et al., 2015) being well established.

Similar structural damage and functional alterations have been reported in the nose and upper airways, with cigarette smoking affecting cilia structure and function of the nasal epithelium (Kuehn et al., 2015; Pagliuca et al., 2015). As result of this, significant dysfunction in nasal mucociliary clearance (MCC) is present (Stanley et al., 1986; Pagliuca et al., 2015). MCC is a major host defense mechanism that protects human airways and lungs against the harmful effects of inhaled toxicants and pathogens. The integration of secretion of airway mucus from goblet cells with synchronized ciliary beating by ciliated epithelial cells provides an efficient defense system (Wanner et al., 1996). Disruption of the MCC may contribute to inflammation and obstruction of the small airways (Cosio et al., 1980), and increased susceptibility to respiratory infections (Konrad et al., 1994: Prescott et al., 1995).

Nasal MCC can be measured with Saccharin test, a non-invasive, well tolerated and simply to perform method that measures mucociliary clearance transit time (MCCTT) (Rodrigues et al., 2019).

Measurement of MCCTT may be exploited as a sensitive functional test for the detection of early changes in respiratory health. A case in point is that of exposure to tobacco smoke. Prolonged MCCTT by Saccharin test has been consistently reported in smokers (Stanley et al., 1986; Pagliuca et al., 2015), with one study showing no significant difference between smokers and never smokers (Nicola et al., 2014). Individual (e.g., cumulative tobacco smoke exposure, passive smoking, occupational history, nasal pathologies, and medications) and environmental factors (e.g., humidity, temperature, and air pollution) (Sherly and Prathibha, 2014) may be accounted for the variability for saccharin test findings. Standardization and reproducibility of the saccharin test are therefore required to minimize its variability and be confident of test results. The issue of test variability is particularly important when investigating subjects with nasal pathologies or with significant exposure to tobacco smoke. Moreover, very little is known about the reversibility of MCC in the smokers who quit smoking. The impairment of the cilia-mucus functional system caused by smoking may be permanent with little possible restoration of MCCTT after smoking abstinence. If this is true, former smokers should exhibit similar MCCTT impairment as reported in current smokers.

Objective of the study is to investigate and compare short- and long-term repeatability of saccharin transit time (STT) among current, former, and never smokers.

## Materials and Methods

### Study Population

The study population consisted of three study groups identified among a pool of subjects who attended a smoking cessation clinic [Centro per la Prevenzione e Cura del Tabagismo of the University of Catania (CPCT)] in the previous 2 years or contacted among hospital staff.

Study group 1 consisted of current smokers, defined as smokers who completed their smoking cessation programs at CPCT in the previous 2 years and were still smoking ≥10 cigarettes per day when contacted for enrollment, with an exhaled carbon monoxide (eCO) level of ≥7 ppm.

Study group 2 was formed of former smokers, defined as quitters of at least 6 months who completed their smoking cessation programs at CPCT in the previous 2 years and were still abstinent when contacted for enrollment, with an eCO level of <7 ppm.

Study group 3 consisted of never smokers, defined as having never smoked or who reported having smoked less than 100 cigarettes in their lifetime (Centers for Disease Control and Prevention [CDCP], 2009). Their eCO had to be <7 ppm to exclude subjects significantly exposed to cigarette smoke or to environmental sources of carbon monoxide (CO).

Current, former and never smokers had to satisfy the following exclusion criteria:

ο Any conditions that could impair cilia-mucus interaction or interfere with MCCTT measurements, such as:
  • Recent (less than 14 days) history of viral infection of the upper respiratory tract.
  • Conditions that may damage nasal mucosa (e.g., chronic rhinosinusitis, infectious rhinitis, allergic rhinitis, atrophic rhinitis, vasomotor rhinitis).
  • Respiratory conditions that may interfere with MCCTT measurements (e.g., COPD, asthma, bronchiectasis, cystic fibrosis).
  • Significant exposure to passive smoking (excludes current smokers).
  • Significant exposure to aerosol emissions from e-cigarettes or heated tobacco products.
  • Significant environmental/occupational exposure to pollution or chemicals (e.g., living in proximity of areas characterized by heavy vehicles traffic, or by presence of industrial fumes; employment in chemical/metallurgy industries).
  • Medications such as pain killers, sleeping pills, antihistamines.
  • Poor individual ability to detect sweetness (i.e., being below the 25 mm mark on the 0–100 mm VAS for sweetness intensity rating).
  • Pregnancy.

The study was approved by the local Ethical Review Board (number 37/2018/PO, Comitato Etico Catania 1. AOU Policlinico–Vittorio Emanuele) and participants gave written informed consent prior to participation in the study.

### Study Design and Study Visits

**FIGURE 1:**
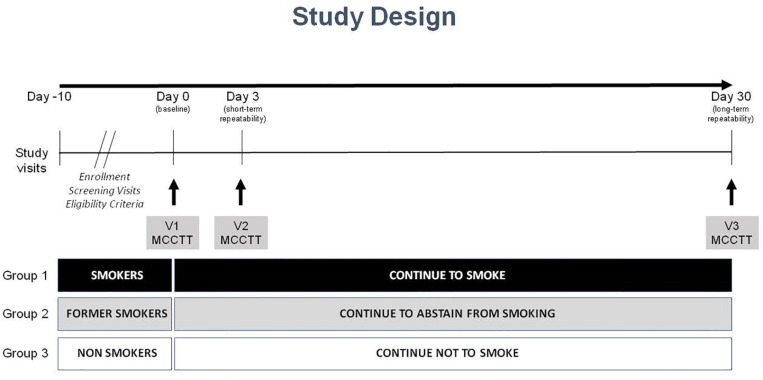
Study design.

This is an observational study with a cross-sectional design to assess MCCTT among three study populations (current, former and never smokers). The study consisted of a total of four morning visits (to avoid the possible influence of circadian rhythms); a screening visit, a baseline visit (V1), and two follow-up visits at day 3 (±1 day) (V2) and at day 30 (±3 days) (V3) (Figure 1). Participants were asked to refrain from drinking coffee/caffeinated drinks for at least 4 h prior to each study visit. Smokers were asked not to smoke for at least 1 h prior to each study visit.

#### Screening Visit

Potential participants attended a screening visit to verify eligibility criteria: socio-demographic data, medical history, medication usage, and smoking/vaping history were noted.

At screening and prior to enrolment, all subjects were tested for exhaled CO and their ability to detect sweetness. Perception of sweetness intensity was rated by using a 0–100 mm VAS. After rinsing the mouth with tap water and wiping the tongue dry with a paper towel, subjects were instructed to smear a saccharine tablet (Mini-sweeteners; Hermesetas; Switzerland) all around the surface of their tongue. They then were asked to rate the intensity of sweetness perception on a 0–100 mm VAS. Sweetness intensity ratings ranged from “*not at all sweet*” (at 0 mm) to “*extremely sweet*” (at 100 mm). Anybody below the 25 mm mark on the VAS was excluded from participation. Eligible subjects were invited to attend the baseline visit.

#### Baseline Visit (Visit 1)

Baseline visits were carried out within 10 days of the screening visit. Eligibility criteria were verified once again.

Before commencing the saccharin test, subjects’ nose was rinsed with warm saline (NaCl 0.9% solution; 2 mls into each nostril). After repeating this procedure twice for each nostril, subjects were then asked to gently blow their nose to remove any excess of fluids (i.e., secretions and saline solution). After nasal washing, subjects were invited to acclimatize at the controlled environmental conditions of the examination room (temperature 21–24°C; relative humidity 30–50%) for at least 45 min, during which BP, HR, oxygen saturation (SpO2) and body mass index (BMI) measurements were performed.

The saccharin test was then carried out (the procedure is detailed in the “Saccharin Test” section below) and baseline transit times recorded. Self-reported nasal and general symptoms were monitored after placing the Saccharin tablet in the nose.

Subjects were instructed to avoid taking medications like pain killers or sleeping pills and invited to attend next study visit (V2), to complete the programmed study assessments/procedures.

#### Day-3 Visit (Visit 2)

Visit 2 was carried out within 3 (±1) days of the baseline visit. Eligibility criteria were verified once again. Nasal lavages and saccharin tests were repeated for short term repeatability. BP, HR, SpO2, and symptoms were noted. Subjects were instructed to avoid taking medications like pain killers or sleeping pills within 5 days of the final study visit (V3) and invited to complete the programmed study assessments/procedures.

#### Day-30 Visit (Visit 3)

The final visit was carried out within 30 (±3) days of the baseline visit. After re-checking eligibility criteria, nasal lavages and saccharin tests were repeated for long term repeatability. BP, HR, SpO2, and symptoms were noted.

### Saccharin Test Method

After nasal washing with warm saline, participants were invited to acclimatize in an examination room optimized for ambient temperature and humidity (i.e., temperature 21–24°C; relative humidity 40–60%). After 45 min acclimatization, participants were invited to slightly raise and tilt the head backward. Whilst illumining a nostril (that indicated by the subject as the one allowing better nasal breathing – the same nostril will be used for all tests providing patency is maintained throughout study visits) with the medical headlight and widening it by using a nasal speculum, the research investigator (or ENT research nurse) identified the small crest that marks the tip of the inferior turbinate. The nipper clasping a saccharin tablet was guided through the speculum and the tablet was gently placed horizontally on the medial face of the inferior turbinate, about 1 cm behind its anterior end. The nipper and nasal speculum were withdrawn paying attention not to trigger any sneezing. Subjects were then invited to return their heads to a straight position and a chronometer was started. Subjects were asked to swallow some saliva a few times every minute until perceiving the “sweet taste” of saccharin. Self-reported nasal and general symptoms were noted at 3 and 10 min after the Saccharin tablet was placed in the nose. Subjects were instructed to avoid to sniff, sneeze, eat, drink, walk, talk, cough, scratch or blow their nose.

### Statistical Analysis

For computing the study sample size, we adopted data from a previous Saccharin test study in smokers indicating a mean MCCTT of 13 min (Ramos et al., 2011). For non-smoker subjects we used data from our Center (unpublished data) indicating a mean MCCTT of 7 min (±2 SD), thus estimating that a sample of 40 subjects for each group was adequate to provide a power greater than 95% with a type-I error (alpha) smaller than 0.01 (1%).

The short-term repeatability of the saccharine test was assessed by linear regression analysis of measurements obtained at V1 and those obtained at V2 for each study group. Likewise, the long-term repeatability was evaluated by linear regression analysis of measurements obtained at V1 and those obtained at V3. Scatter plots of linear regression analyses were generated to visualize repeatability results. Moreover, “Bland and Altman” plots were created to describe the level of agreement between V1 vs. V2 and V3 vs. V1 for each study groups. 1-tailed sample *t* test was also performed to assess the difference from zero of the mean difference between two measurements.

The upper limit of normality (ULN) was calculated by computing the value corresponding to the mean + SD × 1.64 from the distribution curve of the results of the MCCTT measurements in never smokers. Kolmogorov–Smirnov test was performed to assess the data distribution. Categorical data were summarized by counts and percentages; continuously distributed data, with symmetrical distribution, were summarized using the mean [standard deviation (SD)]; continuously distributed data, with skewed distribution, were summarized using the median [inter-quartile range (IQR)]. Study groups comparisons were carried out by Chi-square test, ANOVA and Kruskal–Wallis test for categorical, continuously symmetric and continuously skewed datasets, respectively.

Multiple regression tests were also performed to identify individual variables, including age, gender, BMI, eCO level, pack/years, cig/day, and Fagerstrom Test For Nicotine Dependence (FTND), that may influence the results of the Saccharin test. All analyses were considered significant with a *p* value < 0.05. R version 3.4.3 (2017-11-30) was utilized for data analysis and generation of graphs.

## Results

### Study Participants

**FIGURE 2:**
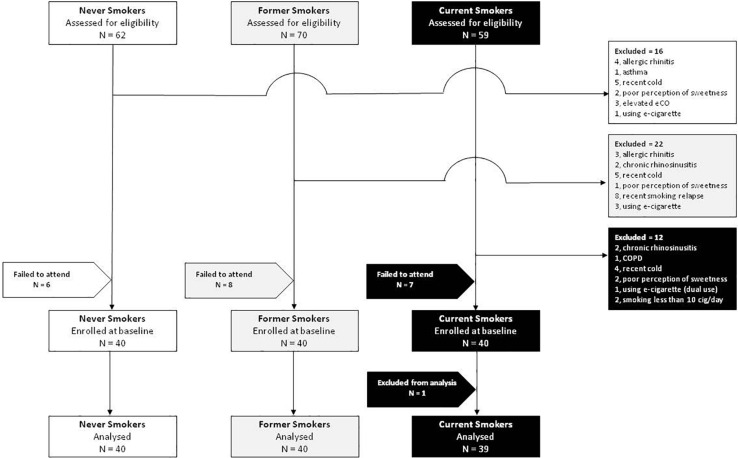
Flow diagram showing the enrollment of subjects into the study.

After screening 191 subjects, 50 were excluded and 21 failed to attend their baseline visit. In total, 120 subjects were enrolled in the study, 40 current, 40 former, and 40 never smokers (Figure 2). Complete analysis on the saccharin test was carried out in 119 subjects (59 F; mean ± SD age of 35.7 ± 13.2 years) (Table 1). One subject from the current smokers study group was not included in the analysis because of an incongruous response to the saccharin test.

**TABLE 1:** Clinical characteristics of study groups.

	Never Smokers	Smokers	Former Smokers	*p* value
Subjects no.	40	39	40	
Age	32.5 (25–41)	31 (24.5–44)	33 (25.75–41.25)	0.909
Female	20/40 (50%)	19/40 (47.5%)	20/40 (50%)	0.991
BMI	23.45 (20.90–25.48)	24.9 (23.4–28.25)	23.95 (20.03–26.55)	0.041
Exhaled CO	3 (2–4)	19 (15–22.5)	2.5 (1.75–5.00)	< 0.0001
Pack/Years	NA	12.5 (6.15–20.35)	15 (5.06–25.35)	0.731*
Cigarettes/day	NA	15 (11–20)	20 (15–25)	0.063*
SBP	114.5 ± 11.03	123.82 ± 11.37	119.85 ± 9.73	< 0.001
DBP	65.68 ± 8.58	75.85 ± 7.12	72.17 ± 8.55	< 0.0001
HR	70.35 ± 9.99	73.15 ± 7.76	74.18 ± 11.66	0.21
SpO2	98 (97.75–98.25)	98 (98–98)	98 (97–98.25)	0.509
FTND	NA	6 (5–7)	NA	

Data are presented as mean ± SD, median (IQR), n/N (%) unless otherwise stated. BMI: body mass index; CO: carbon monoxide; SBP: systolic blood pressure; DBP: diastolic blood pressure; HR: heart rate; SpO2: oxygen saturation; FTND: Fagerstrom Test For Nicotine Dependence. *Comparison analysis carried out only between smoker and former smoker groups.

### MCCTT Repeatability in Never Smokers

**FIGURE 3:**
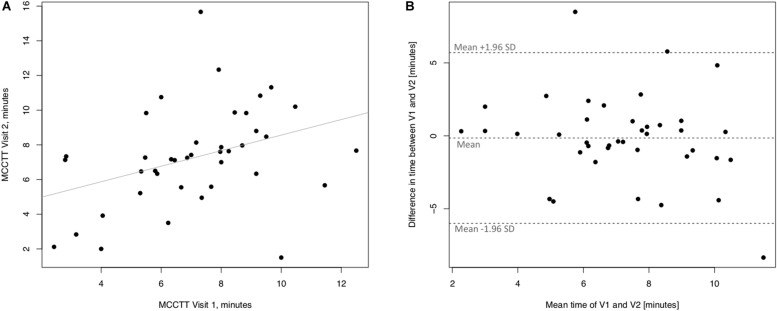
Short-term repeatability (V2 vs. V1) in Never smokers. The left panel **(A)** shows the scatter plot of regression analysis of MCC Transit Time measurements between visit 2 (V2) and visit 1 (V1). The right panel **(B)** shows the difference between the measurements taken at V1 and V2 with respect to the mean in each subject in the Bland Altman plot.

Linear regression analyses performed to assess short-term (3 days) repeatability of saccharin test for never smokers are illustrated in Figure 3. We observed significant regression analysis between V2 and V1 with an R squared = 0.134 and *p* = 0.020 (Figure 3A). Only two subjects had a difference time between V1 and V2 outside the 95% confidence interval (Figure 3B). Moreover, the mean value of the difference between the measurements taken at V1 and V2 (−0.15) was not significantly different from zero (*p* = 0.753).

**FIGURE 4:**
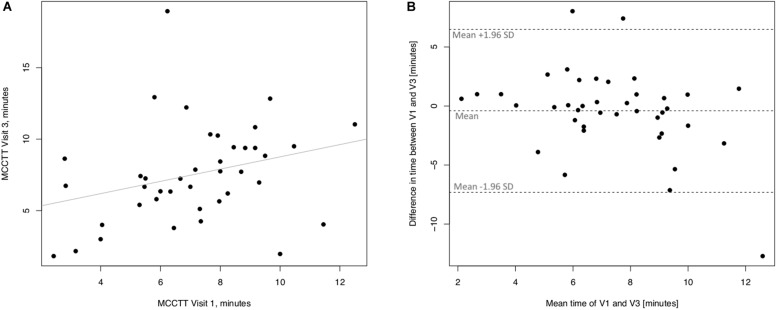
Long-term repeatability (V3 vs. V1) in Never smokers. The left panel **(A)** shows the scatter plot of regression analysis of MCC Transit Time measurements between visit 3 (V3) and visit 1 (V1). The right panel **(B)** shows the difference between the measurements taken at V1 and V3 with respect to the mean in each subject in the Bland Altman plot.

Linear regression analyses performed to assess long-term (30 days) repeatability of saccharin test are showed in Figure 4. The linear regression analysis between V3 and V1 was not significant with a R squared = 0.088 (*p* = 0.063) (Figure 4A). However, only three subjects had a difference time between V1 and V3 outside the 95% confidence interval (Figure 4B). Moreover, the mean value of the difference between the measurements taken at V1 and V3 (−0.405) was not significantly different from zero (*p* = 0.472).

### MCCTT Repeatability in Current Smokers

**FIGURE 5:**
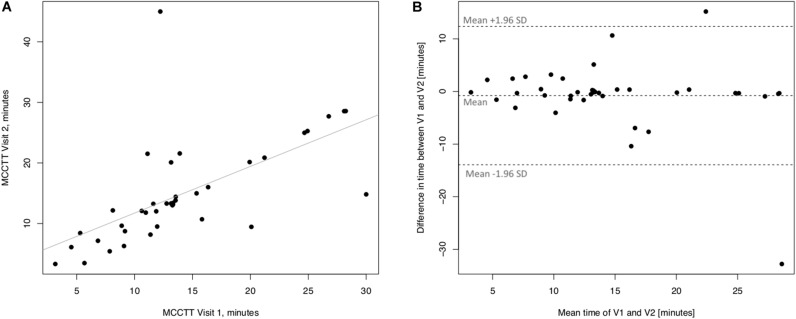
Short-term repeatability (V2 vs. V1) in Current smokers. The left panel **(A)** shows the scatter plot of regression analysis of MCC Transit Time measurements between visit 2 (V2) and visit 1 (V1). The right panel **(B)** shows the difference between the measurements taken at V1 and V2 with respect to the mean in each subject in the Bland Altman plot.

Linear regression analyses performed to assess short-term (3 days) repeatability of saccharin test for current smokers are illustrated in Figure 5. Significant regression analysis was observed between MCCTT at V2 and MCCTT at V1 with an R squared = 0.398 (*p* < 0.0001) (Figure 5A). Only two subjects had a difference time between V1 and V2 outside the 95% confidence interval (Figure 5B). Moreover, the mean value of the difference between the measurements taken at V1 and V2 (−0.781) was not significantly different from zero (*p* = 0.472).

**FIGURE 6:**
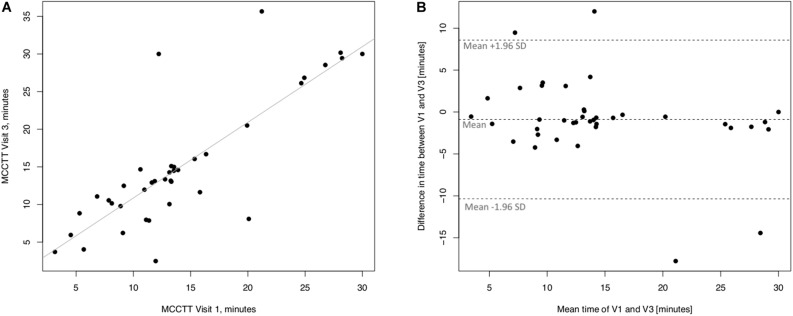
Long-term repeatability (V3 vs. V1) in Current smokers. The left panel **(A)** shows the scatter plot of regression analysis of MCC Transit Time measurements between visit 3 (V3) and visit 1 (V1). The right panel **(B)** shows the difference between the measurements taken at V1 and V3 with respect to the mean in each subject in the Bland Altman plot.

Linear regression analyses performed to assess long-term (30 days) repeatability of saccharin test are showed in Figure 6. The linear regression analysis between V3 and V1 was also significant with a R squared = 0.672 (*p* ≤ 0.0001) (Figure 6A). Four subjects had a difference time between V1 and V3 outside the 95% confidence interval (Figure 6B), and the mean value of the difference between the measurements taken at V1 and V3 (−0.89) was not significantly different from zero (*p* = 0.257).

### MCCTT Repeatability in Former Smokers

**FIGURE 7:**
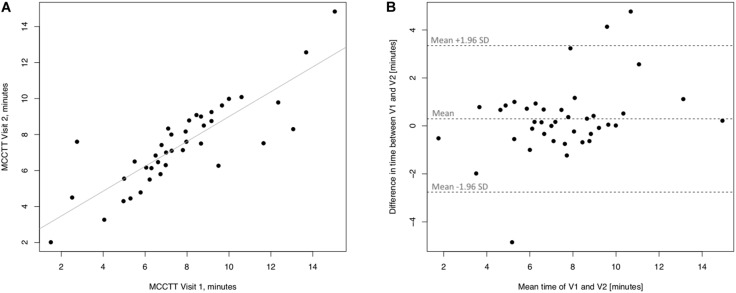
Short-term repeatability (V2 vs. V1) in Former smokers. The left panel **(A)** shows the scatter plot of regression analysis of MCC Transit Time measurements between visit 2 (V2) and visit 1 (V1). The right panel **(B)** shows the difference between the measurements taken at V1 and V2 with respect to the mean in each subject in the Bland Altman plot.

Linear regression analyses performed to assess short-term (3 days) repeatability of saccharin test for former smokers are illustrated in Figure 7. Significant regression analysis was observed between MCTT measured at V2 and MCTT measured at V1 in former smokers with an R squared = 0.714 (*p* < 0.0001) (Figure 7A). Only three subjects had a difference time between V1 and V2 outside the 95% confidence interval (Figure 7B). Also, the mean value of the difference between the measurements taken at V1 and V2 (0.293) was not significantly different from zero (*p* = 0.241).

**FIGURE 8:**
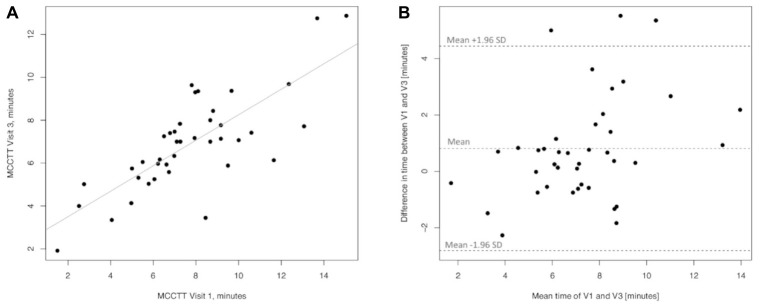
Long-term repeatability (V3 vs. V1) in Former smokers. The left panel **(A)** shows the scatter plot of regression analysis of MCC Transit Time measurements between visit 3 (V3) and visit 1 (V1). The right panel **(B)** shows the difference between the measurements taken at V1 and V3 with respect to the mean in each subject in the Bland Altman plot.

Linear regression analyses performed to assess long-term (30 days) repeatability of saccharin test are showed in Figure 8. The linear regression analysis between MCCTT at V3 and MCCTT at V1 was also significant with a R squared = 0.595 (*p* < 0.0001) (Figure 8A). Only three subjects had a difference time between V1 and V3 outside the 95% confidence interval (Figure 8B). Although the mean value of the difference between the measurements taken at V1 and V3 (0.815) was significantly different from zero (*p* = 0.008), this was very small.

### Multiple Linear Regression Analyses

None of the individual variables investigated (age, gender, BMI, exhaled CO levels, pack/years, cig/day number, and FTND values) had any effect on MCCTT measurements in any of the three study groups.

### Comparison Between Current, Former and Never Smokers

**FIGURE 9:**
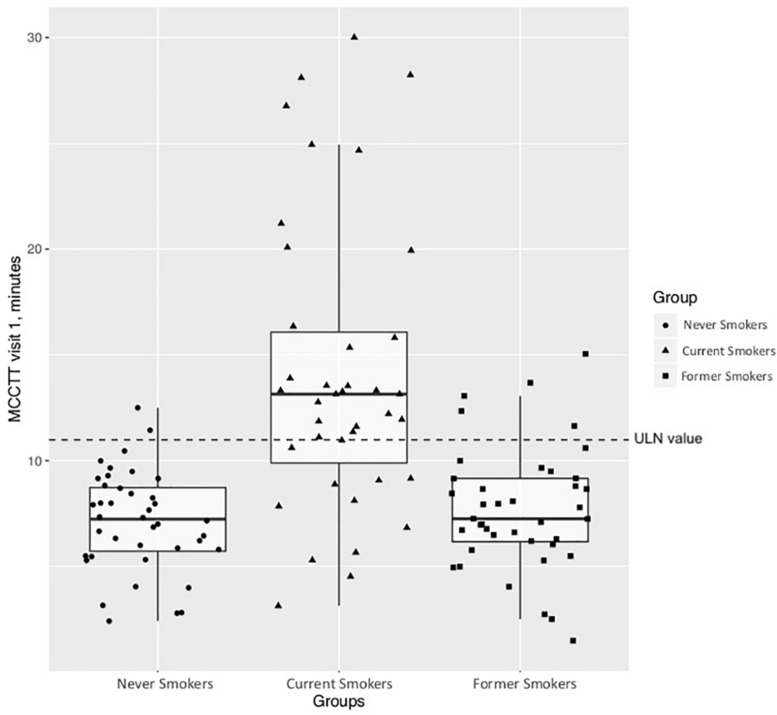
Comparison of MCCTT between Nevers, Current, and Former smokers. The median MCCTT (IQR) values of never smokers and former smokers are similar with a complete overlap of the measurements between the two study groups. On the contrary, the median MCCTT (IQR) value of current smokers is much higher compared to never smokers and former smokers. With much less overlap compared with never smokers and former smokers.

Significant differences in baseline MCCTT were observed among the three study groups (*p* < 0.0001); the median (IQR) MCCTT being 13.15 (10.24–17.25), 7.26 (6.18–9.17), and 7.24 (5.73–8.73) minutes for current, former and never smokers, respectively (Figure 9). MCCTT was significantly higher in current smokers than former and never smokers (*p* value < 0.0001). There was no significant difference between former and never smokers (*p* value = 0.5128).

The calculated ULN of 10.99 min for Saccharin test transit time was used as a cut-off point for abnormal MCCTT measurements. As expected, most of current smokers (27/39; 69.2%) had an MCCTT value above the ULN. Only 12.5% (5/40) former smokers had MCCTT values above the ULN.

### Saccharin Test Tolerability

Side effects were infrequently reported during the course of the Saccharin test and there were no differences among current, former and never smokers. Nasal itch, nasal irritation, and sneezing at 3 min were most commonly reported respectively with 18.4, 15.3, and 4.1% among all participants (average for V1-V2-V3 combined). When present, nasal symptoms were transient and waned immediately thereafter, with no symptoms being reported at 10 min. Moreover, no significant changes in mean (±SD) resting HR, and systolic/diastolic BP were observed.

## Discussion

Study findings show significant repeatability of STT not only when the test was repeated short-term (at day 3) but also long-term (at day 30). High level of repeatability was observed in both current and former smokers. Compared to never smokers, STT was prolonged in current smokers, but not in former smokers. The Saccharin test was very well tolerated by all participants. The MCCTT ULN of 11 min in our study population is well within published threshold discriminating normal people from those with compromised MCC.

This is the first study to investigate STT repeatability in current and former smokers. In both study populations, the linear regression analyses showed significant short-term (V2 vs. V1) and long-term repeatability (V3 vs. V1), despite the low value of R squared. On the other hand, the mean values of differences (V1–V2 and V1–V3) were not significantly different from zero, which means that the first measurement is not affecting the second and the difference does not vary in any systematic way over the range of measurements. We believe that the good reproducibility of MCCTT in this study was due to: (1) meticulous planning and competent conduct of the saccharin test; (2) optimization of environmental conditions of the examination room by close control of ambient temperature and humidity; (3) adequate subject’s preparation (nasal washing, and ambient acclimatization) before beginning saccharin testing; as the presence of excessive secretions may lead to high level of variability of the test, we have introduced nasal lavage with normal saline in the protocol to normalize baseline values (reproducibility data demonstrate that the approach is valid and should be included in any saccharin test protocol) and (4) involvement of a well-trained operators for correct and accurate conduct of the saccharin test. The good reproducibility of STT in current and former smokers is a novel and important finding as it indicates that measurement of MCCTT may be exploited as a reliable and sound biomarker of physiological effect for the detection of early respiratory health changes for clinical research.

Reproducibility for healthy never smokers was modest. Nonetheless, it must be noted that reproducibility (both short and long term) for current smokers and former smokers is very good. Given that most immediate clinical application of the test will not involve healthy never smokers, the modest repeatability in this subjects group has little impact on future clinical application of the test.

Our study confirms two previous observations that STT in current smokers on average doubles that of never smokers (Stanley et al., 1986: Xavier et al., 2013). The large between groups difference was not unexpected in view of the exclusion of light and occasional smokers from participation in the study; low level exposure is known to have little or no effect on MCCTT (Xavier et al., 2013; Nicola et al., 2014). Impairment of the cilia-mucus functional system caused by smoking may take many years to resolve after stopping smoking. In the study of Pagliuca et al., ex-smokers – who abstained on average for 11.7 years – exhibited similar MCCTT as non-smokers. We have also failed to show MCC impairment in former smokers, their STT being similar to never smokers. This was surprising, in view of the fact that former smokers in our study abstained from smoking no longer than 14 months (relatively recent quitters). Therefore, MCCTT restoration after smoking cessation can happen soon after quitting. Nasal mucosa has excellent regeneration potentials and quitting smoking for sufficient periods of time may reverse these deleterious changes; a recent study has shown rapid regeneration of ciliated cells after quitting smoking (Elwany et al., 2020). Prospective studies are required to clarify the time-course of MCCTT restoration after smoking cessation.

The demonstration that impairment of the cilia-mucus functional system caused by smoking can be fully reversed soon after quitting together with good reproducibility results in current and former smokers is clear indication that measurement of STT may be used as a sensitive biomarker of physiological effect for the detection of early respiratory health changes in clinical research. Evaluation of MCCTT changes in smokers undergoing smoking cessation, ex-smokers undergoing relapse prevention studies, and in switching trials of combustion-free nicotine delivery systems (e.g., e-cigarettes, heated tobacco products, new smokeless tobacco products, etc.) is compelling for clinical research.

## Data Availability Statement

The raw data supporting the conclusions of this article will be made available by the authors, without undue reservation.

## Ethics Statement

The studies involving human participants were reviewed and approved by Ethical Review Board (number 37/2018/PO, Comitato Etico Catania 1. AOU Policlinico–Vittorio Emanuele). The patients/participants provided their written informed consent to participate in this study.

## Author Contributions

RE carried out the study and analyzed the data. PC carried out the study, formulated the research question, designed the study, analyzed the data, and wrote the manuscript. FC analyzed the data. MC and AG carried out the study. GC assisted in the correction and revised the manuscript. FB carried out the study was involved in data analysis and corrections. SF was involved in data analysis and formulated the research question. RP formulated the research question, designed the study, analyzed the data, and wrote the manuscript. All the authors approved the final version of the manuscript.

## Conflict of Interest

The authors declare that the research was conducted in the absence of any commercial or financial relationships that could be construed as a potential conflict of interest.

## Abbreviations

BMI: body mass index
CO: carbon monoxide
COPD: chronic obstructive pulmonary disease
CPCT: Centro per la Prevenzione e Cura del Tabagismo of the University of Catania
DBP: diastolic blood pressure
eCO: exhaled carbon monoxide
FTND: Fagerstrom Test For Nicotine Dependence
HR: heart rate
IQR: inter-quartile range
MCC: mucociliary clearance
MCCTT: mucociliary clearance transit time
SBP: systolic blood pressure
SE: standard error
SpO2: oxygen saturation
ULN: upper limit of normality.
